# Effects of short-term warming and nitrogen addition on the quantity and quality of dissolved organic matter in a subtropical *Cunninghamia lanceolata* plantation

**DOI:** 10.1371/journal.pone.0191403

**Published:** 2018-01-23

**Authors:** Xiaochun Yuan, Youtao Si, Weisheng Lin, Jingqing Yang, Zheng Wang, Qiufang Zhang, Wei Qian, Yuehmin Chen, Yusheng Yang

**Affiliations:** 1 Key Laboratory for Subtropical Mountain Ecology, School of Geographical Science, Fujian Normal University, Fuzhou, China; 2 Institute of Geography Science, Fujian Normal University, Fuzhou, China; Tennessee State University, UNITED STATES

## Abstract

Increasing temperature and nitrogen (N) deposition are two large-scale changes projected to occur over the coming decades. The effects of these changes on dissolved organic matter (DOM) are largely unknown. This study aimed to assess the effects of warming and N addition on the quantity and quality of DOM from a subtropical *Cunninghamia lanceolata* plantation. Between 2014 and 2016, soil solutions were collected from 0–15, 15–30, and 30–60 cm depths by using a negative pressure sampling method. The quantity and quality of DOM were measured under six different treatments. The spectra showed that the DOM of the forest soil solution mainly consisted of aromatic protein-like components, microbial degradation products, and negligible amounts of humic-like substances. Warming, N addition, and warming + N addition significantly inhibited the concentration of dissolved organic carbon (DOC) in the surface (0–15 cm) soil solution. Our results suggested that warming reduced the amount of DOM originating from microbes. The decrease in protein and carboxylic acid contents was mostly attributed to the reduction of DOC following N addition. The warming + N addition treatment showed an interactive effect rather than an additive effect. Thus, short-term warming and warming + N addition decreased the quantity of DOM and facilitated the migration of nutrients to deeper soils. Further, N addition increased the complexity of the DOM structure. Hence, the loss of soil nutrients and the rational application of N need to be considered in order to prevent the accumulation of N compounds in soil.

## Introduction

Warming and nitrogen (N) deposition are the two major trends of global climate change. IPCC (2013) reported a 0.78°C increase in the average global surface temperature between 2003 and 2013 compared with that of the 1900–1950 average. With industrialization, the amount of atmospheric N deposition had continued to increase. In 1995, global N deposition had increased to 100 Tg N yr^−1^ and is projected to double by 2050 [1]. China has become the world’s third largest region of N deposition [2]. Climate change alters the biogeochemical cycles of terrestrial ecosystems, such as plant primary productivity [3], microbial community structure [4], soil respiration [5], and N mineralization [6]. Recently, the effects of climate change on dissolved organic matter (DOM) have attracted extensive attention [7–8].

Generally, DOM is considered to be a continuum of organic molecules of different sizes and structures, operationally defined as organic material passing through filters with an effective pore size of 0.45 μm [9]. DOM in the soil solution provides a direct source of nutrients for plant roots and provides energy for the metabolism of microorganisms [9]. In addition, dissolved organic carbon (DOC) and dissolved organic nitrogen (DON) are important C and N pools in forest soil, respectively, which play pivotal roles in biological processes such as N leaching, mineralization, plant absorption, and utilization of N sources [10–11].

The spectral characteristics of DOM help to reveal the spatial and temporal dynamics of nutrient distribution, mobility, and maintenance. UV-Vis spectra and FTIR spectroscopy are commonly used techniques to distinguish and quantify different functional groups in DOM, including both fluorescent and nonfluorescent substances [12–13]. Three-dimensional excitation-emission matrices (EEMs) fluorescence spectroscopy is a promising tool to track DOM. EEMs for DOM characterization are traditionally evaluated by visual inspection. Recently, parallel factor analysis (PARAFAC) techniques are increasingly used to deconvolute complex EEMs into independent fluorescence components [14]. Applications of EEMs, both with and without PARAFAC, have been used in aqueous ecosystems. However, very few studies on soil solutions of forest ecosystems have been reported using EEM-PARAFAC, which primarily focuses on the DOM from soil leaching [15].

Although DOM accounts for less than 5% of soil organic matter (SOM), it has higher activities and plays a major role in nutrient turnover. Warming, N deposition, and other drivers of climate change have the potential to alter the DOM content in soil solution [15–18]. For instance, some studies have shown that warming can promote the production of DOM [19]. Because the increase in temperature accelerated the degradation of SOM [20–21], the process converted complex substances into simple ones [21]. However, Chang et al. found that warming decreased the concentration of DOC in the mineral soil layer [22]. N deposition might cause soil acidification [23], promote nutrient availability in the soil [6], and ultimately affect soil DOM cycling. Recently, a global study showed that the variation in DOC concentrations correlated with soil NH_4_^+^ concentration and C/N ratio [24]. Verstraeten et al. found that the increase in DON in temperate forests is attributed to a series of interrelated abiotic processes, including an increase in pH [25]. Xu et al. conducted an N addition experiment in a mixed forest by using Korean pine and broadleaf at Changbai Mountains, but suggested that N addition decreased the concentration of DOC in soil solutions at 60 cm depth [16]. Conversly, Rappe-George et al. found that the concentration of DOC in soil solution in the O’ horizon was similar between N treatments and control [17]. Thus, the effect of warming and N deposition on DOM remains controversial. Evaluating the structure and dynamics of DOM has become an urgent task for elucidating the effect of global change on DOM. In addition, few global studies have investigated the interaction between warming and N addition, especially in subtropical forests [26–27].

The effects of DOM on C and N cycling might be considerably larger in subtropical forests than in temperate regions [28]. Scientists are increasingly focusing on tropical and subtropical regions [29–30]. Therefore, in this study, the dynamics of DOM were monitored in soil solutions for 2 years. We measured the spectroscopic features of DOM at Sanming Forest Ecosystem and Global Change Research Station in Fujian Province. Our aim was to determine the effects of warming, N addition, and warming + N addition on the quantity and spectral characteristics of DOM in the forest soil solution from a subtropical *Cunninghamia lanceolata* plantation. We hypothesized that warming and the combination of warming and N addition could increase the DOM by increasing the decomposition of SOM. The spectral characteristics of DOM might reveal more detailed structural changes. To the best of our knowledge, this is the first study to investigate the effects of short-term soil warming and N addition on DOM in subtropical forests, which might provide a better understanding of the effects of climate change on the C cycle in forest ecosystems.

## Materials and methods

### Study sites and experimental design

The experiment was conducted at the Fujian Normal University’s Forest Ecosystem and Global Change Research Station in Sanming City in the Fujian Province of China (26°19′ N, 117°36′ E). The average annual precipitation, temperature, and evapotranspiration of the study area are 1670 mm, 19.1°C, and 1585 mm, respectively. The experiment used a randomized complete block factorial design, with warming and N fertilization as fixed factors. There were six treatments (five replicates) in this study, namely, (1) unwarmed and unfertilized (CT); (2) unwarmed and low N (LN); (3) unwarmed and high N (HN); (4) warmed and unfertilized (W); (5) warmed and low N (WLN); and (6) warmed and high N (WHN). There were 30 plots (2 m × 2 m), and the indigenous soil in the plots was replaced, to a depth of 60 cm, with topsoil from the same forest area.

Artificial warming and N addition were initiated in March 2014. Heating cables were used to generate a warmed environment and were buried in a spiral pattern 10 cm below the ground. Soil temperature was significantly increased (5°C) in the warmed plots at the 10 cm depth and measured by temperature sensors (T109; Campbell Scientific Inc., Logan, UT, USA). In order to simulate the shading effects of the heater, “dummy” heaters were used under the control and unwarmed plots. During the same period, we added three N levels (0, 40, and 80 kg N ha^-1^ year^-1^) as ammonium nitrate (NH_4_NO_3_) according to local conditions. NH_4_NO_3_ was divided into 12 doses at regular intervals. Complete details of the experimental design have been described previously by Zhang et al. [31]. The daily mean air temperature and daily precipitation at the study plot during the study period are shown in S1 Fig.

### Soil solution sampling and analysis

In each experimental plot, soil suction samplers (suction lysimeter, SM32) were installed at 15, 30, and 60 cm soil depths in April 2014. Soil solutions were sampled monthly from May 2014 to April 2016. The soil solution was collected using the negative pressure method [32], and then filtered through a 0.45 μm membrane in the laboratory and kept refrigerated (<4°C) within a week until analysis. DOC was measured using a total organic carbon (TOC) analyzer (TOC-L CPH/CPN, Japan). Dissolved total N (DTN) and total dissolved inorganic N (NO_3_^—^N and NH_4_^+^-N) were analyzed using a continuous flow analyzer (Skalar san^++^, Netherlands). Dissolved organic N (DON) was equal to the difference between DTN and total dissolved inorganic N.

### Soil sampling and analysis

In April 2016, we used a 5-cm soil core to collect the soils (0–15, 15–30, and 30–60 cm depths) from each plot from five random points. The soil samples were immediately transported to the laboratory and stored at 4°C within a week until analyses. The soil was cleared of roots and all organic debris. Fractions of air-dried soil samples were ground and passed through a 2 mm sieve for analyzing soil pH, soil organic C, and total N. The soil pH was determined using a pH meter with a soil:water ratio of 1:2.5. Soil organic C and soil total N were measured by using a CN auto analyzer (Elementar Vario MAX, Germany). Another fraction of fresh soil was used to measure soil microbial biomass carbon (MBC) and microbial biomass nitrogen (MBN) using the chloroform fumigation-extraction method [33]. Finally, MBC was analyzed using a total organic carbon analyzer (TOC-VCPH/CPN, Japan), whereas MBN was analyzed using a continuous flow analyzer (Skalar san++, Netherlands).

### Spectral analysis

The values of Specific UV Absorbance, SUVA254 and SUVA260, have been widely used as indices for the abundance of aromatic carbon and hydrophobic components of DOM, respectively [34–36]. UV spectra were obtained on a UV-2450 (Shimadzu, Japan) in quartz cuvettes with a 1 cm path length, and Milli-Q water was used as a reference. Specific UV absorbance (SUVA254 and SUVA260) was calculated as the absorbance at 254 and 260 nm normalized to the DOC concentration, respectively, according to the following equation [34–35]:

SUVA=(UV/DOCconcentration)×100 (1)

Fluorescence emission spectra were obtained using a Hitachi F-7000 fluorescence spectrophotometer (λ_ex_ 254 nm, slit 10 nm, λ_em_ 300–480 nm, slit 10 nm, and scan speed 1200 nm min^-1^). The humification index (HIX) was defined as the ratio of the peak area of the emission spectrum in the Σ435–480 nm quarter to that of the Σ300–345 nm quarter at a fixed excitation wavelength of 254 nm [37]. HIX characterizes the degree of DOM humification, and higher HIX values indicate that DOM has undergone more humification and has a more complex structure [37].

The fluorescence index (FI) was calculated as the ratio of emission intensity at 470 to that at 520 nm, at a fixed excitation wavelength of 370 nm [38–39]. The index of autochthonous contribution (BIX) was calculated as the ratio of emission intensity at 380 to that at 430 nm, at a fixed excitation wavelength of 310 nm [40]. The freshness index (β:α) was calculated as the ratio of emission intensity at 380 nm divided by the maximum intensity between 420 and 435 nm, at a fixed excitation wavelength of 310 nm [39].

Excitation emission matrix (EEM) fluorescence was measured using a Hitachi F-7000 fluorescence spectrophotometer with scanning emission spectra from 250 to 600 nm (every 2 nm), at an excitation of 200–450 nm (every 5 nm). Excitation/emission slit widths were 5 nm and the scan speed was 2400 nm min^-1^ at 700 V photomultiplier. Samples were diluted to DOC < 2 mg L^-1^ prior to measurement to avoid inner filter effects. The spectra of all samples were blank corrected using Milli-Q water and normalized with the water Raman signal (integral emission intensity of 370–420 nm at excitation of 350 nm) [41]. The coefficient of the water Raman signal variation was approximately 5% over daily measurement.

One milligram of the freeze-dried DOM sample was ground and mixed thoroughly with 400 mg of oven-dried KBr powder of analytical grade (Merck, DAC, USA). The mixture was then compressed into a transparent disk. The infrared spectra were recorded using a Nicolet Magna FTIR 550 double-beam infrared spectrophotometer.

### PARAFAC modeling

EEMs only express the fluorescence information of a sample; however, EEMs combined with PARAFAC can detect the main fluorescence characteristics of samples and provide a comprehensive analysis of their main components [42]. PARAFAC, a multiway data analysis method, is used to decompose complex fluorescence EEMs into chemically meaningful spectral components [43]. Briefly, the data array is reduced to trilinear terms by minimizing the sum of squares of the residuals using the following equation:

$$x_{ijk}=\sum_{f=1}^{F}a_{if}(b_{jf}\times c_{kf})+\varepsilon_{ijk}$$ (2)

where *x*_*ijk*_ expresents the fluorescence intensity of sample *i* measured at emission wavelength *j* and excitation wavelength *k*. *a*_*if*_ is proportional to the concentration of each sample component. The products of the relative emission spectra (*b*_*jf*_) and excitation spectra (*c*_*kf*_) produce a fluorescence matrix of the component *f* at unit concentration. The final term *ε*_*ijk*_ indicates the residual error containing instrument noise and/or un-modeled variations [44]. In this study, PARAFAC decomposition was analyzed using MATLAB with DOMFluor toolbox downloaded from Chemometrics site at the University of Copenhagen.

### Statistical analysis

The effects of treatments on DOC, DON, DTN, NO_3_^-^, NH_4_^+^, and DOC:DON ratio in the soil solution were assessed using repeated measures analysis of variance. Multivariate analysis of variance and least significant differences were used to test the effects of the treatments on soil properties for the entire study period. All statistical analyses were conducted using SPSS 20.0. Diagrams were drawn using Origin 9.0. PARAFAC analysis of DOM components was performed using MATLAB R2014a and DOMFluor software packages. The spectral characteristics and output of DOM components were drawn using MATLAB.

## Results

### DOM concentration in soil solution

**Fig 1:**
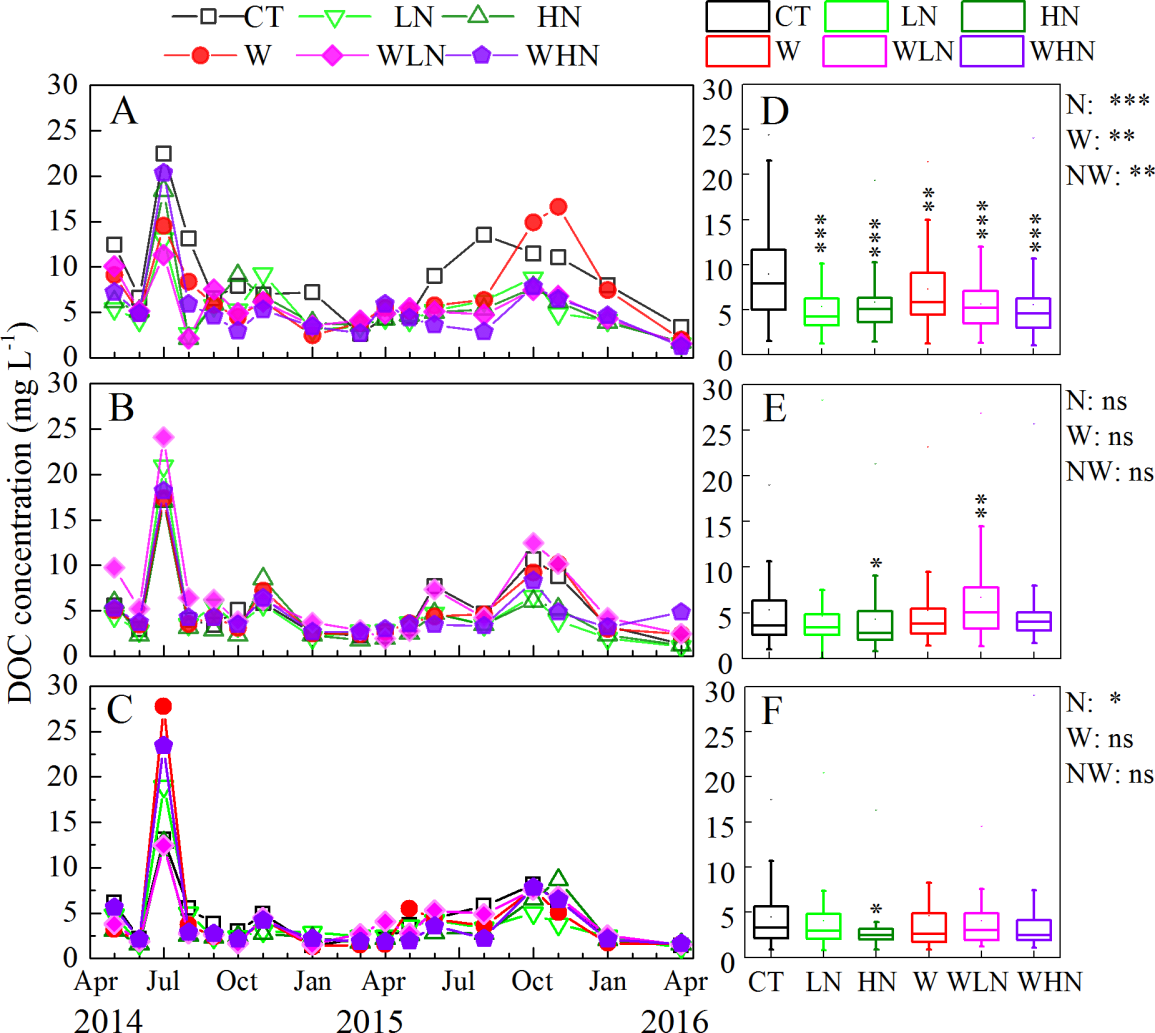
**Monthly dynamics of DOC concentration (A–C) and a box-plot (D–F) for soil solution with warming and nitrogen addition treatments from April 2014 to April 2016.** The 0–15 cm layer (A, D), 15–30 cm layer (B, E), 30–60 cm layer (C, F). N: nitrogen addition effect; W: warming effect; WN: interaction effect of warming and nitrogen addition; ns: no significant differences; * indicates that differences were significant at the 0.05 level (two tailed); ** indicates that differences were significant at the 0.01 level (two tailed); *** indicates that differences were significant at the 0.001 levels (two tailed).

A significant decreasing trend in DOC concentration (*p* < 0.05) in surface soil solution was observed with warming, N addition, and warming + N addition compared with that of CT (Fig 1A and 1D). The effect of N addition on DOC was more significant than that of warming, and LN and HN decreased by 39.7% and 34.2%, respectively (Fig 1D). The decreasing proportions of DOC in the surface layers of WLN and WHN, 37.2% and 37.8%, respectively, were comparable to those observed with N addition. In deeper soil layers, the inhibitory effect of HN on DOC remained significant. While warming increased DOC concentration in the 15–30 cm soil layer, WLN increased significantly (Fig 1E). The median DOC concentration in the 30–60 cm soil layer ranged from 0 to 5 mg L^-1^, which was significantly lower than that in the surface layer (5–10 mg L^-1^) (Fig 1F). Monthly dynamic fluctuations in all layers under all different treatments followed similar patterns, with peaks appearing in summer and autumn (Fig 1).

**Fig 2:**
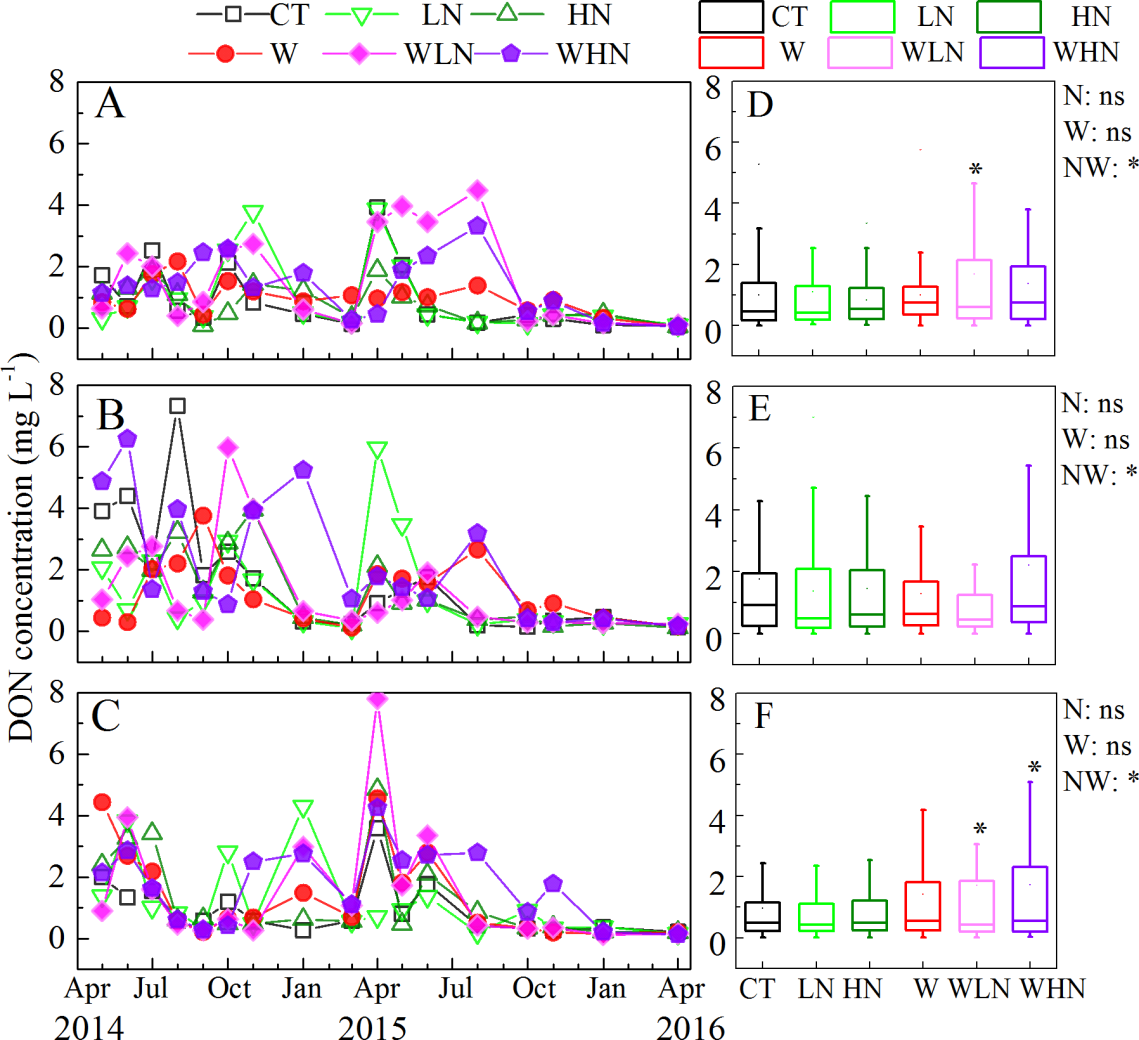
**Monthly dynamics of DON concentration (A–C) and box-plot (D–F) in soil solution with different treatments from April 2014 to April 2016.** The 0–15 cm layer (A, D), 15–30 cm layer (B, E), and 30–60 cm layer (C, F). N: nitrogen addition effect; W: warming effect; WN: interaction effect of warming and nitrogen addition; ns: no significant differences; * indicates that differences were significant at the 0.05 level (two tailed).

The DON concentration of WLN in the 0–15 and 30–60 cm soil layers was 1.7 and 1.8 times, respectively, higher than that of CT (Fig 2D and 2F). The monthly dynamics of DON concentration showed frequent fluctuations. After 1 year, a decreasing trend in the DON concentration was observed with warming + N addition. From October 2015, the DON concentration was about 1 mg L^-1^ and remained stable thereafter (Fig 2).

A non-significant increasing trend of N forms was observed with N addition treatments such as DTN, NO_3_^-^, and NH_4_^+^ (Table 1). Correspondingly, a significant increasing trend in DTN and NO_3_^-^ concentration was observed with warming and warming + N addition, with only an increasing trend observed for NH_4_^+^ (Table 1). Under the warming treatment, DTN and NO_3_^-^ increased by 1.2–1.9 and 1.5–2.6 times, respectively, compared with those of CT, and the DTN and NO_3_^-^ concentrations with warming + N addition were twice as those of CT (Table 1). Warming and N addition alone had no significant effect on DOC:DON ratio, whereas warming + N addition significantly decreased the DOC:DON ratio in the surface layer (Table 1).

**Table 1:** Concentrations (mg L^-1^) of DTN, NO_3_^-^, and NH_4_^+^ in soil solution under different treatments.

Indicators	Soil layers (cm)	CT	LN	HN	W	WLN	WHN	N	W	NW
DTN	0–15	6.06(0.74)	6.66(0.42)	7.35(1.10)	10.73(0.51)***	9.32(1.69)***	11.13(1.28)***	ns	***	***
	15–30	5.31(1.14)	5.42(1.37)	7.14(1.82)	10.05(2.03)***	9.13(1.68)**	15.91(2.68)***	ns	**	**
	30–60	5.88(0.85)	5.78(0.83)	6.32(1.19)	7.04(1.12)	7.47(2.22)	10.17(2.16)***	ns	***	***
NO_3_^-^	0–15	3.86(0.39)	3.93(0.51)	5.89(0.59)**	10.01(0.69)***	7.34(1.06)***	11.58(1.29)***	ns	***	***
	15–30	3.68(0.15)	4.18(1.08)	5.31(0.66)	7.96(1.29)***	6.85(1.31)**	11.19(2.91)***	ns	**	**
	30–60	3.99(0.37)	4.69(0.55)	4.51(0.8)	6.14(1.42)**	7.85(1.75)***	9.94(1.59)***	ns	**	***
NH_4_^+^	0–15	0.18(0.07)	0.30(0.14)*	0.17(0.02)	0.31(0.06)*	0.22(0.02)	0.27(0.08)	ns	*	ns
	15–30	0.21(0.05)	0.24(0.05)	0.22(0.06)	0.25(0.04)	0.29(0.17)	0.37(0.09)**	ns	ns	*
	30–60	0.31(0.33)	0.24(0.04)	0.32(0.26)	0.14(0.04)	0.23(0.11)	0.15(0.05)	ns	ns	ns
DOC: DON	0–15	20.42(2.88)	16.9(3.22)	19.67(3.38)	16.32(3.28)	10.06(2.43)***	13.44(5.73)**	ns	ns	***
	15–30	13.07(2.44)	11.73(4.04)	9.43(1.99)*	10.51(1.77)	13.55(1.86)	8.98(1.45)*	ns	ns	ns
	30–60	11.64(2.77)	10.28(3.34)	10.46(1.96)	9.73(1.99)	11.43(1.35)	11.11(2.60)	ns	ns	ns

N: nitrogen addition effect; W: warming effect; WN: interaction effect of warming and nitrogen addition; ns: no significant differences.

* indicates that differences were significant at the 0.05 level (two tailed).

** indicates that differences were significant at the 0.01 level (two tailed).

*** indicates that differences were significant at the 0.001 levels (two tailed).

### Spectroscopy index of DOM

SUVA254 and SUVA260 increased with N addition (Table 2). The humic degree of DOM in the soil solution was generally lower, and HIX values were all less than 1 (Table 2). In contrast, N addition increased DOM humification, and the HIX values of LN and HN were twice higher than that of CT. BIX and FI can indicate the origins of DOM. A slight difference was noted in the BIX and FI values between treatments: FI values were generally greater than 2, whereas BIX values were close to 1 (Table 2). The rate of DOM production was relatively slow, and 2% to 5% proportion was reflected in the freshness index (β:α), which indicated the contribution of recently produced DOM. The Fn(355) value in the surface soil layer decreased after 2 years of warming and N addition (Table 2).

**Table 2:** Spectroscopy characteristic index values of DOM in the soil solution, including SUVA254, SUVA260, HIX, Fn(355), β:α, FI, and BIX.

Soil layer (cm)	Treatments	SUVA254	SUVA260	HIX	Fn(355)	β:α	FI	BIX
0–15	CT	3.04(0.15)	2.70(0.33)	0.40	262.50	0.05	2.42	0.97
	LN	4.13(0.21)	3.54(0.26)	0.81	173.80	0.04	2.38	1.00
	HN	4.57(0.18)	4.04(0.32)	0.88	179.70	0.03	2.26	1.00
	W	4.02(0.26)	3.10(0.09)	0.46	206.60	0.04	2.44	0.98
	WLN	4.62(0.14)	3.73(0.16)	0.66	231.70	0.04	2.33	0.90
	WHN	4.64(0.13)	3.52(0.13)	0.73	151.10	0.03	2.34	1.08
15–30	CT	4.93(0.35)	4.25(0.23)	0.84	180.00	0.03	2.34	0.92
	LN	3.99(0.26)	3.55(0.13)	0.77	137.40	0.03	2.15	1.08
	HN	2.18(0.22)	1.79(0.20)	0.67	104.30	0.03	1.88	1.14
	W	2.88(0.16)	2.21(0.23)	0.42	268.40	0.05	2.77	0.95
	WLN	3.10(0.08)	2.72(0.09)	0.14	241.30	0.04	2.42	0.96
	WHN	1.48(0.09)	1.58(0.06)	0.13	157.80	0.03	2.65	0.97
30–60	CT	1.86(0.14)	1.53(0.08)	0.51	137.30	0.02	1.80	0.87
	LN	4.17(0.15)	3.61(0.23)	0.61	129.00	0.02	2.22	0.95
	HN	2.56(0.08)	1.81(0.18)	0.72	165.60	0.04	1.99	0.90
	W	3.41(0.07)	2.52(0.15)	0.54	165.30	0.03	2.38	1.05
	WLN	2.61(0.09)	2.15(0.08)	0.73	181.20	0.03	2.55	0.91
	WHN	3.40(0.21)	2.55(0.17)	0.85	193.20	0.04	2.36	0.97

### UV-Vis spectroscopy

**Fig 3:**
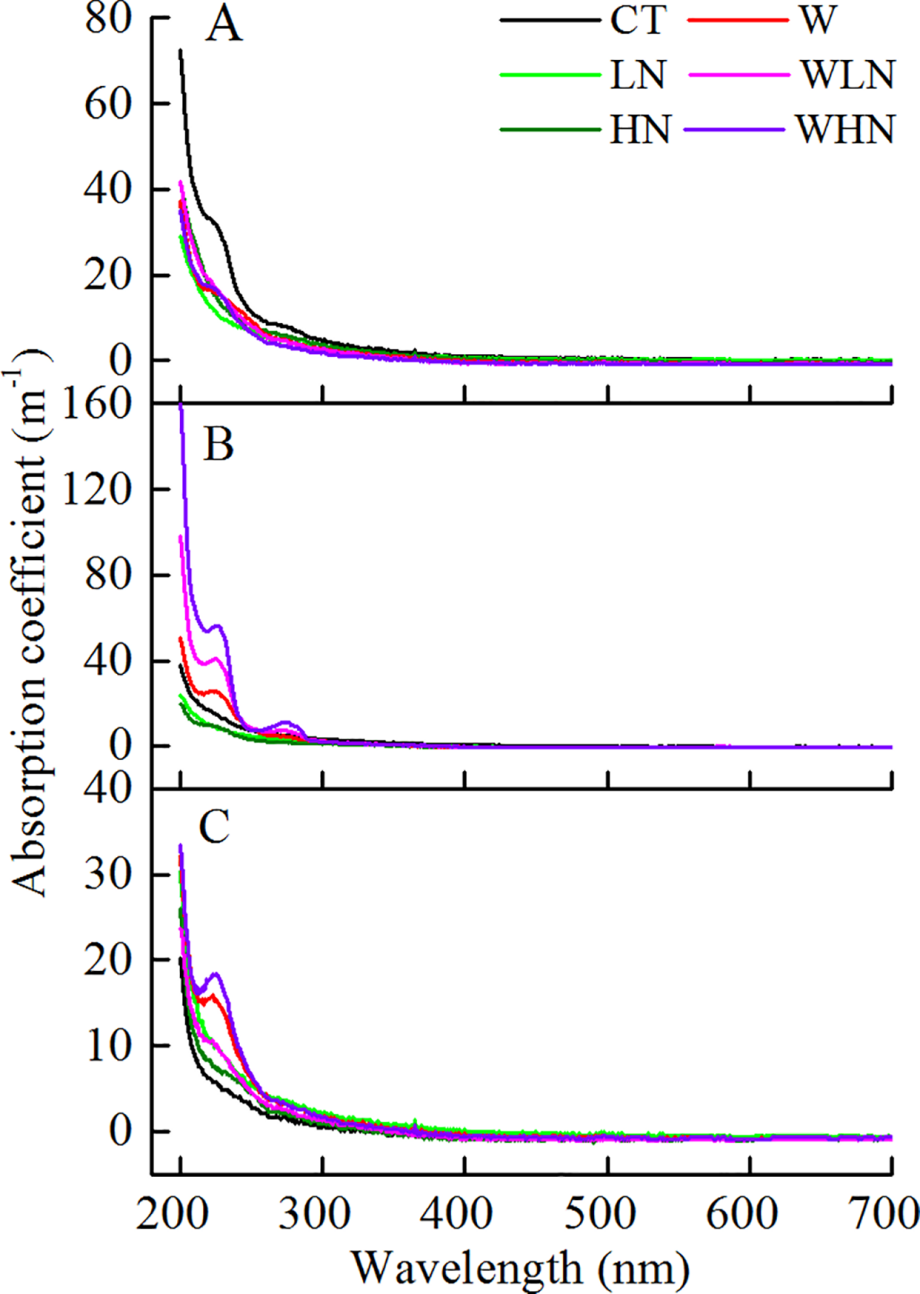
UV-Vis spectra of DOM in the soil solution under different treatments. The 0–15 cm layer (A), 15–30 cm layer (B), and 30–60 cm layer (C). Data on the chemical properties of DOM in soil solution originating from a sample obtained by mixing five soil solution samples in each treatment from April 2016.

The UV-Vis spectra of DOM from the soil solution under different treatments are shown in Fig 3. There was a strong absorption peak in the 210–250 nm wavelength range for each treatment and a weak absorption band in the 250–300 nm wavelength range. The absorbance of each treatment in the 0–15 cm surface layer was lower than that of CT (Fig 3A). With increasing depth, the absorbance of WLN and WHN became significantly higher than that of CT, and WHN was always the highest among all treatments below the 15-cm soil layer (Fig 3B and 3C).

### Infrared spectroscopy

**Fig 4:**
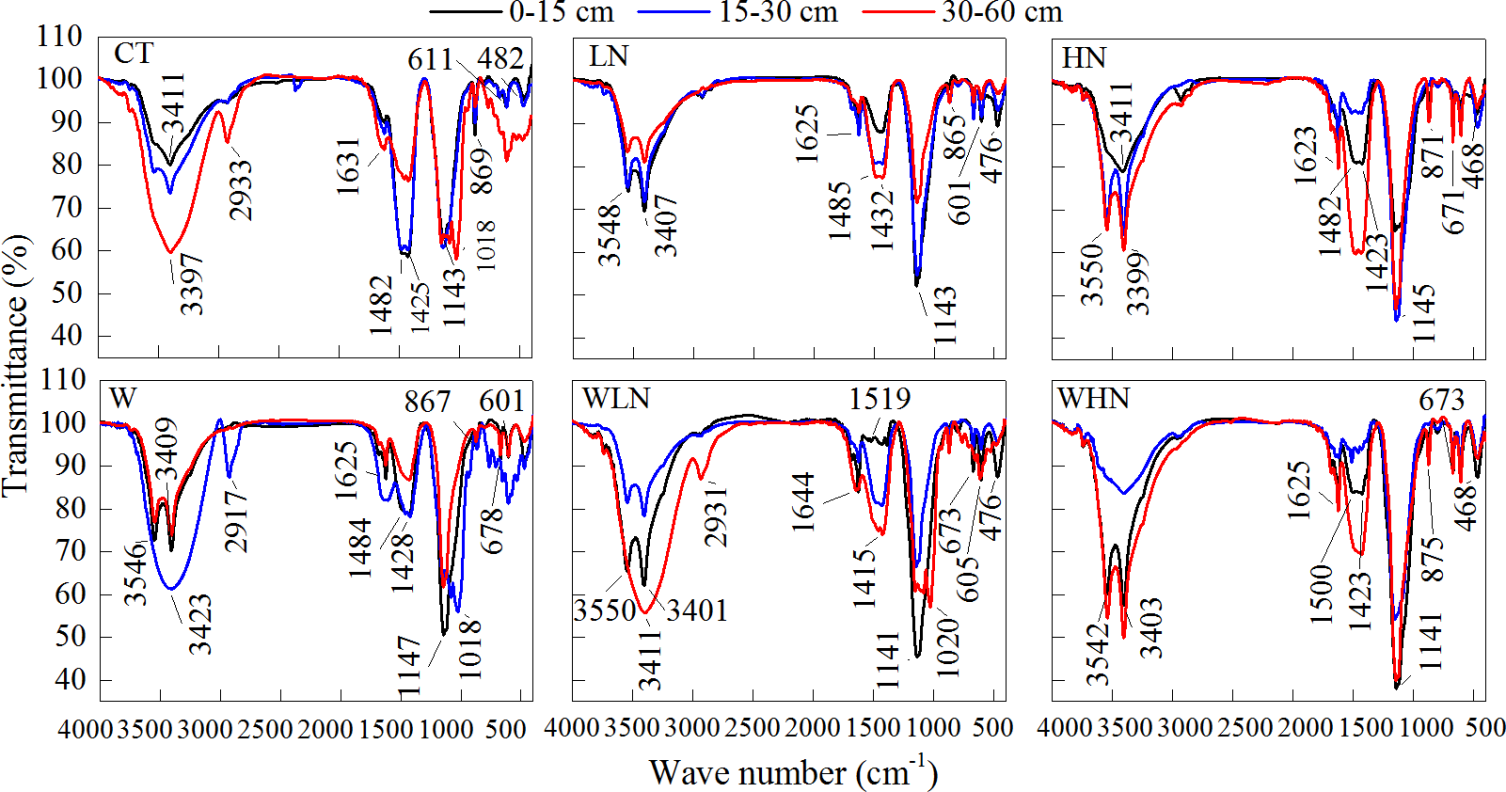
Infrared spectra of DOM in soil solution from different treatments.

The infrared absorption of DOM mainly existed in six regions. Three high intensity absorption bands were observed: the A band (3550–3400 cm^-1^), D band (1550–1300 cm^-1^), and E band (1260–1000 cm^-1^) (Fig 4; S1 Table). The control treatment showed a strong and wide absorption peak at the A band, and the peak transformed into two sharp and strong absorption peaks after warming or N addition. The change was more obvious with the warming + N addition treatment. A significant reduction in the peak at the D band could be observed with both N addition and warming + N addition treatments, and the absorption of DOM in the surface layer was less than 10%. The E band represented high-intensity absorption, which is attributed to the C-O stretching of polysaccharides, alcohols, and carboxylic acids. The peak of E band was further strengthened with warming and warming + N addition, and the absorption of the latter was 50%–60%. In deeper soil, the infrared peaks remained in the same positions.

### Three-dimensional fluorescence

**Fig 5:**
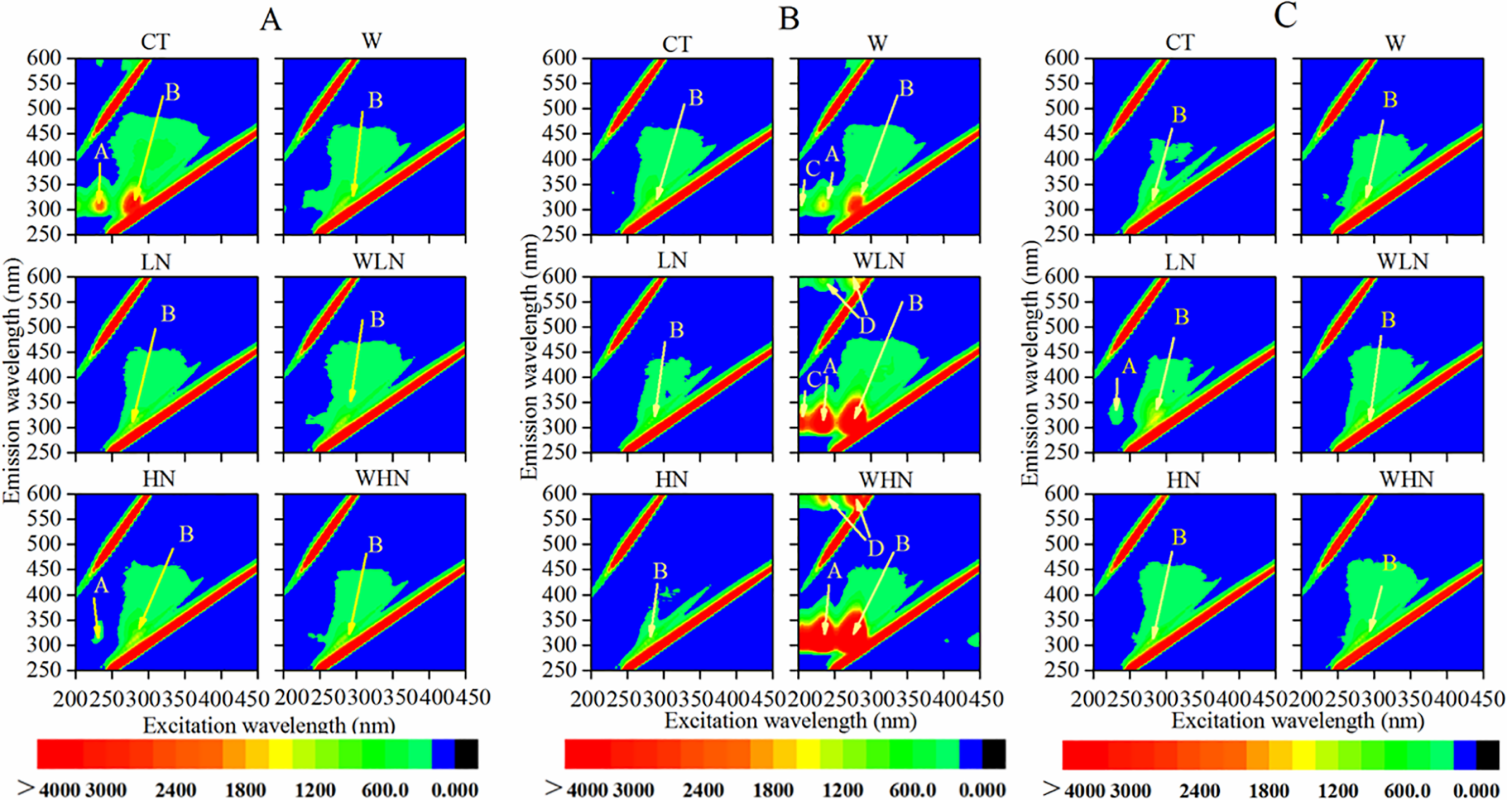
Three-dimensional fluorescence spectrum of DOM under different treatments. The 0–15 cm layer (A), 15–30 cm layer (B), and 30–60 cm layer (C).

The spectrum was divided into five regions based on the fluorescence regional integration method proposed by Chen et al. [45] (S2 Table). The DOM fluorescent peak was mainly located in two regions, namely, an aromatic protein-like fluorescence peak A in Zone I (Ex/Em = 250–280/290–380 nm) and a soluble microbial byproduct-like material fluorescence peak B in Zone IV (Ex/Em = 250–280/290–380 nm) (Fig 5). Fluorescence peak A (Ex/Em = 225/300 nm) belongs to the low-excitatory wavelength of tyrosine. Following 2 years of warming and N addition, compared with CT, peak A in the 0–15 cm soil layer disappeared, with the exception of HN, and peak B also decreased (Fig 5A). The intensities of peaks A and B were higher in the 15–30 cm soil layer than in the surface layer. However, two new peaks (peaks C and D) appeared with warming and warming + N addition. Further, only peak B was observed with CT and N addition treatments (Fig 5B). At soil depths of 30–60 cm, no differences were noted between the test and control (Fig 5C).

### EMMs-PARAFAC

**Fig 6:**
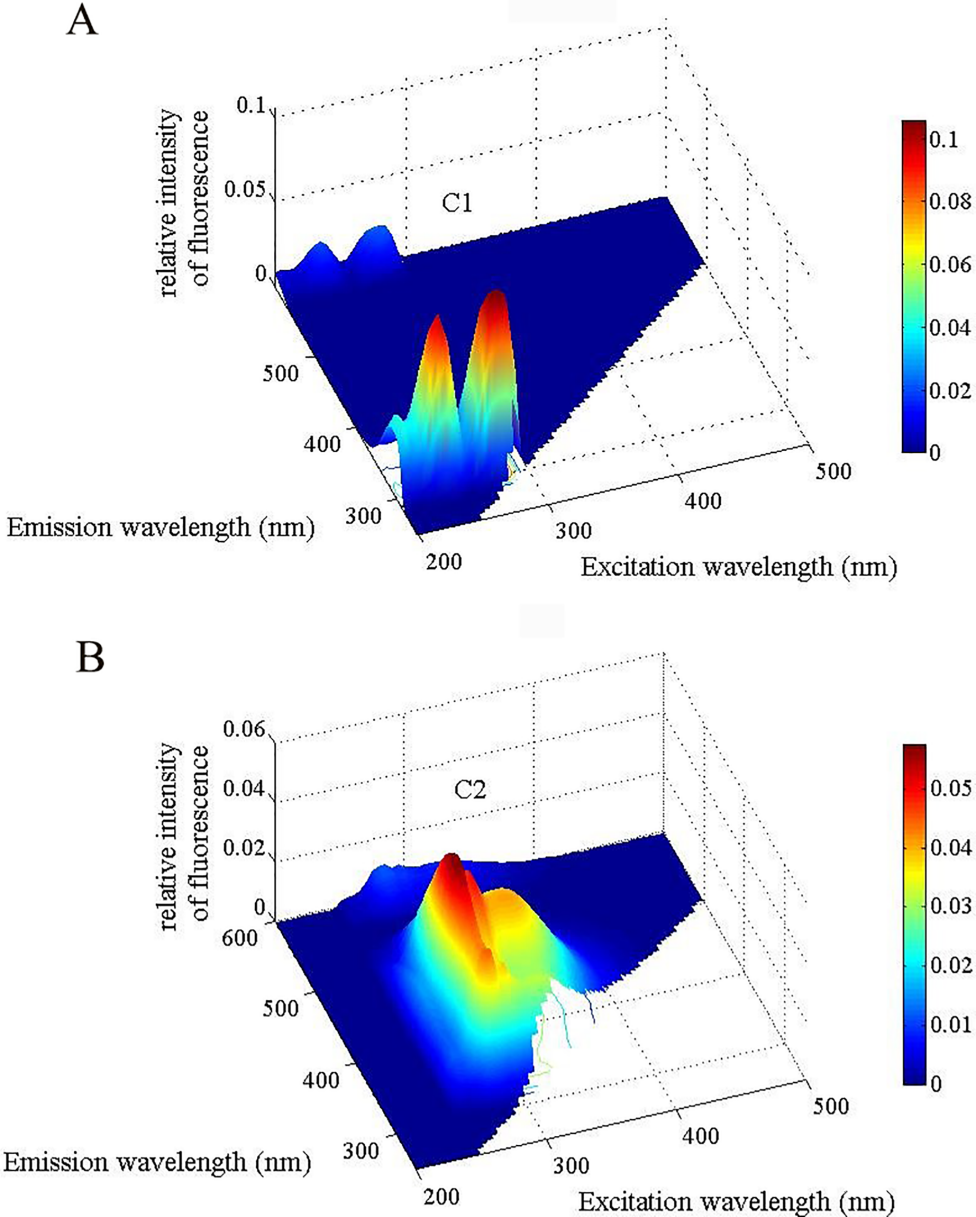
EEM contours of the two components of DOM identified by PARAFAC analysis. (A) C1 is a protein-like component with Ex/Em = <280/300–310 nm; (B) C2 is a humic-like component with Ex/Em = 280–325/400–420.

The results of EMMs-PARAFAC showed that DOM mainly consisted of C1 and C2 components (Fig 6). C1 is a protein-like component with Ex/Em = <280/300–310 nm, where a part of C1 (Ex/Em = 230/300–310 nm) is associated with low excitation-like tyrosine; the other part of C1 (Ex/Em = 275/300–310 nm) is generated by soluble microbial byproduct-like materials. C2 is attributed to the humic-like substances of Ex/Em = 280–325/400–420 [39, 42, 46]. The fluorescence intensity of C1 is higher than that of C2.

## Discussion

### Properties of EMMs-PARAFAC in the DOM of forest soil solution

EEM-PARAFAC is widely applied in various fields, especially in lake ecosystems [47–48]. For example, some studies have shown that protein-like components in the DOM of natural water are mainly produced by microbial degradation [49]. Fu et al. revealed two types each of humic-like fluorescence and protein-like fluorescence in the pore water of Lake Erhai sediments; most of the protein-like components were aromatic structures such as tryptophan and tyrosine [50]. Recently, a study in agricultural soils showed that three fluorescence components, including UVC humic-like substances, UVA humic-like substances, and tyrosine-like materials, were identified in the soil DOM [42]. Our results obtained using PARAFAC analysis showed that the composition of the DOM of the forest soil solution was similar to those of freshwater and agricultural soil, which mainly included protein-like components (C1: Ex/Em = 230(275)/300 nm) and humic-like substances (C2: Ex/Em = 280–325/400–420 nm) (Fig 5), but without UVC humic-like substances. In addition, FI values greater than 2 and BIX values close to 1 were observed with all treatments (Table 2), which could also indicate that microbial degradation was a primary source of DOM in this study. Furthermore, the results of EMMs-PARAFAC showed that humic-like components C2 (Ex/Em = 280–325/400–420 nm) existed in the DOM (Fig 6), which is similar to fulvic acid, with high molecular weight and aromatic intensity and a more complex structure [51].

### The effect of warming on DOM

Temperature directly affects the rates of many biological and chemical reactions in the ecosystems and regulates water, nutrient flow, and ecosystem energy [52]. The production and utilization of DOM are affected by temperature. Warming has also been reported to increase DOC leaching in some studies [20–22]. However, contrary to our hypotheses, significant decrease in DOC was noted in the surface layer (Fig 1A and 1D). This result was consistent with a phenomenon, in which DOM content is lower in tropical sites with high temperature compared with that in boreal and temperate sites, owing to the high production in the tropics offset by high decomposition rates [24]. Previous studies have shown that the loss and utilization of DOM in the soil solution can be attributed to the mineralization and nitrification of the soil [53–55]. We found that warming significantly increased inorganic N (DTN, NO_3_^-^, and NH_4_^+^) (Table 1). These results are in line with those of many studies suggesting that warming increases the soil net N mineralization [53]. Conversly, warming could reduce the amount of DOM originating from microbes and inhibit the decomposition of SOM by reducing the microbial biomass [21, 56–59]. In our study, warming indeed decreased the microbial biomass (S3 Table). The result of three-dimensional fluorescence revealed that peak B (representing microbial metabolites) in the 0–15 cm soil layer weakened with warming (Fig 5A). These results confirmed that the microbial source of DOM tended to decrease, and warming partly inhibited the decomposition of SOM.

However, the concentration of DOC increased in the 15–30 cm layer corresponding to the warming treatment (Fig 1D). The three-dimensional fluorescence spectrum also showed that peak A reappeared and peak B was enhanced in the 15–30 cm layer, whereas no peak was observed for the 15–30 cm soil layer in CT. The fluorescence intensity was also lower than that of the surface layer (Fig 5B), which could be attributed to the loss of surface proteins and other nutrients through warming. Moreover, non-significant influences of DOC and DON were observed in the bottom soil. This was consistent with the findings of Kane et al., who attributed this to the influence of warming on vegetation, the resulting interactions with root-associated microorganisms, and root activity [60].

### The effect of N addition on DOM

Short-term N addition in our experimental area decreased the DOC in the soil solution (Fig 1). This result can be explained by the following three reasons: (1) inhibition of soil respiration, promotion of N accumulation, and reduction of the C:N ratio [61]; (2) the influence of N on microbial activity leading to insoluble organic matter, which is difficult to degrade; and (3) stimulation of plant growth, thereby increasing plant absorption of DOC and other nutrients in the soil solution. The effects of N addition on DOC and DON were different. N addition significantly elevated the content of NO_3_^-^ (Table 1).

In terms of spectroscopy, numerous chromophores or auxochrome might dominate the molecular structure of DOM; auxochrome can move the absorption peaks of the chromogenic groups in the molecules to long wavelengths [62]. The UV absorbance of the K band with N addition was lower than that with CT (Fig 3); this suggested the π→π* orbital transition under N addition condition. FTIR analysis showed a significant decrease in the absorption rates of the C band (1644–1625 cm^-1^) and D band (1550–1300 cm^-1^) (Fig 4), and the protein-like component of DOM significantly decreased in the 0–15 cm soil layer (Fig 5A). The results indicated a decrease or transformation of the carboxylic acid and amino acid contents [63–66]. Further, many substances disappeared with the reduction in DOC, which confirmed that the decrease in protein and carboxylic acid contents was mostly attributed to the reduction of DOC following N addition. LN and HN in the 3550–3400 cm^-1^ appeared with two sharp and strong peaks of absorbance (Fig 5), which belonged to the stretching vibration of N-H, indicating the formation of amines containing RNH_2_. In addition, the HIX, SUVA254, and SUVA260 showed significant increasing trends following N addition in the soil solution (Table 2), which indicated that N addition increased the levels of aromatic compounds, condensed aromatic rings, and macromolecular compounds, and thus increased the complexity of these compounds. We expected that the complexity of its structure might be attributed to the increase of RNH_2_ in amines. Overall, short-term N addition might exacerbate the risk of losing protein-like components in the surface layer. However, the long-term accumulation of N might also lead to the massive accumulation of non-biodegradable substances and result in difficulty in the utilization of soil nutrients.

### The effects of warming and N addition on DOM

The DOC concentration decreased in the 0–15 cm soil layer, but significantly increased in the 15–30 cm soil layer (Fig 1D and 1E) with warming + N addition. The interaction between warming and N addition was significant at enhancing the concentration of DON (Fig 2). Further, aromatic compounds and protein-like components significantly increased in the UV spectrum. In addition, a new humic-like component (peak D) appeared in the 15–30 cm soil layer (Fig 5B), which indicated that the interaction between warming and N addition promoted the humification of substances and increased the degree of DOM humification. Conversely, warming can enhance the effect of N addition on the aminolytic reaction of the carboxyl compounds and promote the formation of RNH_2_ contained in amines. Single-factor responses can be misleading because of the interactions between factors and indirect effects such as increased N availability owing to temperature-induced decomposition [67]. In addition, the interaction effects were mainly attributed to warming, whereas the DOC concentration dropped considerably with warming + N addition mainly owing to the effect of N addition. Therefore, N deposition and climate warming played prominent roles in nutrient cycling.

## Conclusions

The increase in warming and N addition has led to many ecological problems, which have attracted the widespread attention of scientists and the public. The DOM in soil solution mainly consists of protein-like components from microbial degradation. The quantity and quality of DOM in subtropical soil solution were altered with warming + N addition after 2 years. (1) Warming decreased and inhibited DOC in the surface soil solution possibly by reducing related enzyme activities and microbial biomass, promoting soil nitrification and mineralization, and thereby inhibiting the decomposition and transformation of organic matter. (2) N addition reduced the content of DOC. However, the degree of humification, aromaticity, and hydrophobicity (HIX, SUVA254, and SUVA260) were increased under N addition, indicating that short-term N deposition was beneficial for the maintenance of soil fertility. (3) The effects of warming + N addition were interactive rather than additive, and the effect was mainly attributed to warming.

## Supporting information

S1 Fig Daily average air temperature and precipitation in the study area (June 2014–May 2015). (TIF)

S1 Table The main attribution of infrared absorption peaks. (DOCX)

S2 Table The main attributes of three-dimensional fluorescence peaks. (DOCX)

S3 Table The physicochemical properties of each soil layer. (DOCX)
