# Supplementary material for: Effects of short-term warming and nitrogen addition on the quantity and quality of dissolved organic matter in a subtropical Cunninghamia lanceolata plantation
Source: PLoS One. 2018 Jan 23;13(1):e0191403. doi: 10.1371/journal.pone.0191403 (PMC5779672; doi:10.1371/journal.pone.0191403)
Supplement: S1 Table — (DOCX) [file pone.0191403.s002.docx]

**S1 Table. The main attribution of infrared absorption peaks.**

| Spectral region | Wavelength (cm^-1^) | Main attribution |
| --- | --- | --- |
| A | 3600–3300 | -OH stretching of alcohol and phenol groups, stretching vibration of N-H |
| B | 3000–2850 | aliphatic C-H stretching of CH_3_/CH_2_ groups |
| C | 1650–1600 | a mixture of C=O stretching of carboxylates/amides; C=C stretching of aromatic and the COO- antisymmetric stretching of organic carboxylate groups |
| D | 1550–1300 | Symmetrical bending vibration of N-H; vibration of C-H in aliphatic structures |
| E | 1260–1000 | C-O stretching of hydroxyl and ether bonds (typical of carbohydrate linkages) |
| F | 880–605 | O-H deformation of carboxylic acid compounds or aromatic C-H vibrations. |
