# Supplementary material for: Effects of short-term warming and nitrogen addition on the quantity and quality of dissolved organic matter in a subtropical Cunninghamia lanceolata plantation
Source: PLoS One. 2018 Jan 23;13(1):e0191403. doi: 10.1371/journal.pone.0191403 (PMC5779672; doi:10.1371/journal.pone.0191403)
Supplement: S2 Table — (DOCX) [file pone.0191403.s003.docx]

**S2 Table. The main attributes of three-dimensional fluorescence peaks.**

| The region of fluorescence spectrum | Ex/Em | Fluorophore |
| --- | --- | --- |
| I area | 200–250/280–330 nm | Tryptophan, tyrosine, phenylalanine, and other types of aromatic protein-like components |
| II area | 200–250/330–380 nm | Protein-like components |
| III area | 200–250/380–450 nm | Fulvic-like matter |
| IV area | 250–280/280–380 nm | Soluble microorganism metabolites |
| V area | ＞250/380–550 nm | Humic-like components |
