# Supplementary material for: Effects of short-term warming and nitrogen addition on the quantity and quality of dissolved organic matter in a subtropical Cunninghamia lanceolata plantation
Source: PLoS One. 2018 Jan 23;13(1):e0191403. doi: 10.1371/journal.pone.0191403 (PMC5779672; doi:10.1371/journal.pone.0191403)
Supplement: S3 Table — (DOCX) [file pone.0191403.s004.docx]

**S3 Table. The physicochemical properties of each soil layer.**

| Soil layers  (cm) | Treatments | pH | SOC | TN | C/N | MBC | MBN | MBC/MBN |
| --- | --- | --- | --- | --- | --- | --- | --- | --- |
|  |  |  | g kg^-1^ | g kg^-1^ |  | mg kg^-1^ | mg kg^-1^ |  |
| 0–15 | CT | 4.74(0.10) | 11.98(1.66) | 1.25(0.14) | 9.52(0.36) | 212.85(42.64) | 20.09(2.31) | 11.42(3.57) |
|  | LN | 4.76(0.04) | 10.88(0.51) | 1.19(0.04) | 9.26(0.51) | 146.35(25.32)** | 17.05(1.47) | 9.29(2.23) |
|  | HN | 4.74(0.15) | 11.07(1.40) | 1.24(0.14) | 9.01(0.58)* | 164.80(26.86)* | 17.65(4.3) | 9.60(2.03) |
|  | W | 4.75(0.10) | 9.91(1.31)* | 1.08(0.13)* | 9.05(0.21) | 148.41(31.08)** | 11.62(2.37)*** | 13.38(2.93) |
|  | WLN | 4.77(0.11) | 10.84(1.42) | 1.15(0.12) | 9.34(0.30) | 100.90(25.21)*** | 14.26(2.3)** | 7.03(0.67)** |
|  | WHN | 4.53(0.11)** | 11.18(1.25) | 1.19(0.11) | 9.32(0.26) | 117.23(10.7)*** | 10.90(1.34)*** | 10.60(1.62) |
| 15–30 | CT | 5.28(0.02) | 3.39(0.26) | 0.57(0.07) | 6.13(0.80) | 78.37(20.45) | 6.38(1.88) | 19.82(10.96) |
|  | LN | 5.36(0.09) | 3.26(0.40) | 0.60(0.04) | 5.53(0.65) | 66.60(36.81) | 6.66(2.96) | 16.61(9.67) |
|  | HN | 5.26(0.06) | 3.80(0.39) | 0.64(0.07) | 6.26(0.60) | 46.55(13.19)* | 8.25(1.5) | 6.31(1.38)** |
|  | W | 5.21(0.06) | 3.33(0.31) | 0.57(0.06) | 5.85(0.60) | 30.73(3.36)** | 8.11(3.24) | 5.52(3.35)** |
|  | WLN | 5.21(0.14) | 3.43(0.55) | 0.58(0.06) | 5.9(0.40) | 43.77(18.33)* | 6.29(1.9) | 8.83(4.04)* |
|  | WHN | 5.14(0.17)* | 4.50(0.65)* | 0.70(0.08)* | 6.46(0.68) | 36.14(21.5)** | 5.41(1.33) | 9.91(6.20)* |
| 30–60 | CT | 5.37(0.09) | 3.01(0.27) | 0.58(0.02) | 5.23(0.24) | 60.09(16.26) | 5.62(2.02) | 25.60(13.54) |
|  | LN | 5.42(0.30) | 2.97(0.81) | 0.59(0.07) | 5.06(0.78) | 70.53(17.5) | 6.37(2.21) | 14.41(3.47)* |
|  | HN | 5.32(0.10) | 3.21(0.38) | 0.62(0.10) | 5.38(0.60) | 22.12(10.08)*** | 4.93(2.25) | 7.02(2.60)*** |
|  | W | 5.28(0.15) | 3.32(0.54) | 0.58(0.04) | 5.82(0.85) | 28.55(6.35)** | 11.73(3.84)** | 2.41(0.49)*** |
|  | WLN | 5.33(0.10) | 3.26(0.26) | 0.57(0.02) | 5.71(0.56) | 72.35(13.53) | 9.16(6.39) | 11.35(5.70)** |
|  | WHN | 5.09(0.22)* | 3.79(0.57)** | 0.93(0.08)*** | 4.41(0.42)* | 27.53(9.19)*** | 3.58(0.63) | 11.62(3.43)** |

* indicates that differences were significant at the 0.05 level (two tailed); ** indicates that differences were significant at the 0.01 levels (two tailed).
